# Redirection of Epithelial Immune Responses by Short-Chain Fatty Acids through Inhibition of Histone Deacetylases

**DOI:** 10.3389/fimmu.2015.00554

**Published:** 2015-11-03

**Authors:** May Young Lin, Marcel R. de Zoete, Jos P. M. van Putten, Karin Strijbis

**Affiliations:** ^1^Department of Infectious Diseases and Immunology, Utrecht University, Utrecht, Netherlands

**Keywords:** SCFAs, butyrate, toll-like receptors, TLR5, flagellin, NF-κB, histone acetylation, HDAC

## Abstract

Short-chain fatty acids (SCFAs) are products of microbial fermentation that are important for intestinal epithelial health. Here, we describe that SCFAs have rapid and reversible effects on toll-like receptor (TLR) responses in epithelial cells. Incubation of HEK293 or HeLa epithelial cells with the SCFAs butyrate or propionate at physiological concentrations enhanced NF-κB activation induced by TLR5, TLR2/1, TLR4, and TLR9 agonists. NF-κB activation in response to tumor necrosis factor α (TNFα) was also increased by SCFAs. Comparative transcript analysis of HT-29 colon epithelial cells revealed that SCFAs enhanced TLR5-induced transcription of TNFα but dampened or even abolished the TLR5-mediated induction of IL-8 and monocyte chemotactic protein 1. SCFAs are known inhibitors of histone deacetylases (HDACs). Butyrate or propionate caused a rapid increase in histone acetylation in epithelial cells, similar to the small molecule HDAC inhibitor trichostatin A (TSA). TSA also mimicked the effects of SCFAs on TLR–NF-κB responses. This study shows that bacterial SCFAs rapidly alter the epigenetic state of host cells resulting in redirection of the innate immune response and selective reprograming of cytokine/chemokine expression.

## Introduction

The intestinal microbiota is essential for fermentation of complex carbohydrates. As a result of this process, the microbiota produces a multitude of metabolites that can easily cross the mucus barrier and exert local and systemic regulatory functions. Such metabolites have been shown to play a role in the development of inflammatory bowel diseases (IBD) and even impact brain function (1). One group of bacterial metabolites with pleiotropic effects on host immune and energy state are short-chain fatty acids (SCFAs). SCFAs are the major end products of bacterial degradation of soluble fiber in the large intestine, with acetate (C2), propionate (C3), and butyrate (C4) as the main SCFAs produced during carbohydrate and amino acid fermentation (2). Butyrate serves as the major energy source for colonic epithelial cells, which metabolize 70–90% of total butyrate in the gut (3). The major butyrate-producing bacteria are a phylogenetically diverse group of Gram-positive Firmicutes, in specific *Clostridia spp*. (4). The physiological concentrations of SCFAs in the lumen of the large intestine can range from 1 to 25 mM, while plasma concentrations are in the micrometer range (5, 6).

Short-chain fatty acids have important health-promoting effects, for example, by stimulating intestinal gluconeogenesis via gut–brain neuronal circuits resulting in metabolic benefits for the host (7). SCFAs also exert local anti-inflammatory effects and patients with colitis benefit from increased dietary fiber or SCFA intake, or butyrate enemas (8, 9). At the cellular level, SCFAs impact proliferation and immune responses in a cell-specific manner. For example, butyrate promotes proliferation and survival of normal epithelial cells, but induces apoptosis through G1 or G2-M arrest in cancerous cells (10). In colonic epithelial cell lines, butyrate incubation reduces neutrophil migration toward Caco-2 cells but increases migration toward HT-29 cells (11). Because of their pleiotropic and cell-type-dependent effects, the mechanisms of action of SCFAs are poorly understood.

Toll-like receptors (TLRs) are abundantly expressed in the gut and regionally or spatially arranged according to their specific functions (12). TLR5 is highly expressed in the colon (13) where it recognizes flagellin of most motile bacteria and induces protective innate and adaptive immune responses (14). Other TLRs present in the intestine include TLR4 that recognizes Gram-negative lipopolysaccharides (LPS), and TLR2 that senses bacterial lipoproteins and lipoteichoic acid. Sensing of microbial components by TLRs induces activation of the pro-inflammatory transcription factor NF-κB, the release of cytokines and chemokines, and activation of adaptive immune responses (15, 16).

Because both bacterial metabolites and TLR activation are important for intestinal homeostasis, we here assessed the combined effect of SCFAs on the TLR response in intestinal epithelial cells. Most of the previous studies, investigating the effects of SCFAs on cellular processes, focus on long-term effects, for example, overnight or 24 h after treatment. Since SCFAs have profound effects on cell differentiation and proliferation, and these effects could mask the direct effects that SCFAs may have on epithelial immune responses, we choose a short exposure to SCFAs in the majority of our studies. These conditions also more accurately mimic the rapidly increased SCFA levels that occur in the intestine after eating. Our data show that butyrate and propionate rapidly and reversibly redirect the nature of TLR-induced innate immune responses. We provide evidence that these effects are most likely caused by SCFA-induced alterations in histone acetylation through inhibition of histone deacetylase enzymes.

## Materials and Methods

### Cell Culture

HEK293 cells, stably expressing TLR5, were provided by Dr. B. van der Burg (Hubrecht Laboratory Utrecht, The Netherlands). HeLa 57A cells stably transfected with a NF-κB-luciferase reporter system (17) were provided by Dr. R. T. Hay (Institute of Biomolecular Sciences, University of St. Andrews, Scotland). The HT-29 cell line was purchased from American Type Culture Collection (ATCC, Rockville MD, USA). HEK293, HeLa 57A, and HT-29 cells were routinely propagated in T25 flasks in DMEM supplemented with 5%, 5%, and 10% FCS, respectively. All cell lines were cultured at 37^o^C and 5% CO_2_.

### Reagents

Ultrapure LPS and native flagellin from *Salmonella enterica* serovar Enteriditis strain 90-13-706 (*S. enteriditis*) were isolated as described previously (18). The synthetic tripalmitoylated lipopeptide Pam_3_CSK_4_ (N-Palmitoyl-S-[2,3-bis(palmitoyloxy)-(2RS)-propyl]-[R]-cysteinyl-[S]-seryl-[S]-lysyl-[S]-lysyl-[S]-lysyl-[S]-lysine trihydrochloride), and CpG ODN 2006 were purchased from Invivogen (Toulouse, France). The sodium salts of butyrate [sodium butyrate (Na-Bu)] and propionate [sodium propionate (Na-Pro)] and Trichostatin A (TSA) were purchased from Sigma-Aldrich (Zwijndrecht, The Netherlands). Tumor necrosis factor α (TNFα) was purchased from BD Biosciences (Breda, The Netherlands). The expression plasmids pTracer-TLR1, pTracer-TLR2, pTracer-CD14, and pFlag-TLR5 were constructed as previously described (18, 19). In every transfection, the pTK-LacZ vector was used for the normalization of transfection efficiency. The expression vectors pUNO-TLR4, pUNO-MD2, and pUNO-TLR9 were purchased from Invivogen. All TLRs and their co-receptors were of human origin.

### Cell Transfection and Stimulation

HeLa 57A and HEK293 cells were grown in 48-well tissue culture plates (Corning, Schiphol-Rijk, The Netherlands) in culture medium up to 50–60% confluency. Transient transfection with the plasmids carrying the indicated genes was performed with FuGENE 6 (Roche Diagnostics, Almere, The Netherlands) or FuGENE HD (Promega, Leiden, The Netherlands) according to the manufacturer’s instructions. A 3:1 ratio of FuGENE:DNA was used. The total amount of plasmid DNA per transfection was 250 ng/well. For transfection of HT-29 cells, adherent HT-29 cells at a confluence of ~80–90% were trypsinized and re-plated in 48-well tissue culture plates. Directly after plating, cells were transiently transfected with ExGen 500 (Fermentas GmbH, Germany) according to the manufacturer’s instructions. A 6:1 ratio of ExGen 500:DNA was used. The total amount of DNA used per transfection was 500 ng/well. Forty-eight hours post-transfection, the medium was refreshed with new culture medium (including FCS). Cells were pre-treated for 30 min with 10 mM Na-Bu, 10 mM Na-Pro or 2 μM TSA or left untreated. After the pre-incubation period, the medium was not refreshed prior to stimulation with the TLR agonists or TNFα unless indicated otherwise. Pre-treated or untreated cells were stimulated for 5 h with the TLR ligands or TNFα.

### NF-κB-Luciferase Assays

The NF-κB-luciferase reporter system that was used to quantify the NF-κB activation following TLR stimulation was described previously (20). In brief, cells were washed twice with Dulbecco’s PBS and 100 μl of Reporter Lysis Buffer (Promega, Leiden, The Netherlands) was added to each well after which the plate was stored o/n at −80^o^C. Luciferase activity was measured by mixing 50 μl of Luciferase Assay Reagent (Promega) to 20 μl of defrosted cell lysate followed by direct measurement in a luminometer (TD-20/20, Turner Designs, Sunnyvale, CA, USA). The β-galactosidase enzyme assay (Promega) was performed by adding 50 μl of cell lysate to 50 μl of 2× β-galactosidase assay buffer as indicated by the manufacturer’s instructions. Luciferase values were normalized against the β-galactosidase values to obtain relative luciferase units (RLU). Results are expressed as percentage of maximal response of TLR-transfected cells with its cognate ligand. In experiments that included TNFα, TNFα addition was considered the maximum response.

### HDAC Assay

HDAC activity was measured using the HDAC-Glo assay (Promega) according to the manufacturer’s instructions. Cells were incubated with SCFA or TSA at the indicated concentrations for 30 min or 5 h, transferred to an eppendorf tube and lysed in assay buffer with 1% Triton X-100. In a separate experiment, SCFA and TSA were added to the assay mixture after cell lysis. The assay was performed in 96-well format. Protein lysate was added in the linear range of the assay together with 50 μl of assay reagent for a total reaction volume of 100 μl. The assay was incubated at room temperature for 20 min after which luminescence was measured using a Fluorostar plate reader (GE Healthcare).

### RNA Isolation and Quantitative RT-PCR

Total RNA was extracted from HT-29 cells with RNA-Bee (Bio-Connect BV, Huissen, The Netherlands). Isolated RNA was treated with DNase I (Fermentas) to remove remaining DNA. RNA yield was quantified using a NanoDrop ND-1000 spectrophotometer (Thermo Scientific, Rockford, IL, USA). RNA transcript levels were assessed by quantitative real time PCR (qRT-PCR) using the Lightcycler 480 System (Roche, Woerden, The Netherlands). For quantitative RT-PCR of human IL-8 and β-actin, previously described probe and primer pairs were used (18). All primer sequences are listed in Table 1. Primers were labeled with the reporter dye 6-carboxy-fluorescein (FAM) and the quencher tetramethyl-6-carboxyrhodamine (TAMRA). The RT-PCR with probes was performed with the One Step RT-PCR Master-Mix kit for Probe Assays (Eurogentec) with 50 ng of RNA per reaction and 0.8 μM of primers and probes. Real-time cycler conditions were as follows: 30 min at 48^o^C, 10 min at 95^o^C followed by 40 cycles of 15 s at 95^o^C, and 1 min at 60^o^C. Quantitative RT-PCR of TLR5, monocyte chemotactic protein 1 (MCP-1) and TNFα was performed using SYBR Green dye. Primer Express software version 2.0 (Abi Prism, Applied Biosystems) was used to design cytokine primer sets that amplify 50–80 bp fragments. The qRT-PCR was performed with 500 ng of RNA, 1 μM of primers and the Brilliant III Ultra-Fast SYBR Green qRT-PCR kit (Agilent Technologies) according to the manufacturer’s instructions. Real-time cycler conditions were as follows: 10 min at 50°C, 3 min at 95°C, and 45 cycles at 95°C for 5 s and 60°C for 10 s. Melting curves were produced by increasing the temperature to 95°C for 5 s, then 1 min at 65°C followed by slowly increasing the temperature to 97°C. Transcript levels were corrected to β-actin, and fold expression levels of gene of interests were calculated by their Ct value. Unstimulated condition: ΔCt control = Ct target gene control − Ct β-actin control. Stimulated condition: ΔCt treated = Ct target gene treated − Ct β-actin treated. ΔCt(control) − ΔCt(treated) values = ΔΔCt, the fold change in mRNA = 2^ΔΔCt^ (21).

**Table 1:** **Quantitative RT-PCR primers and probes used in this study**.

Gene	Primer/probe	Sequence 5**′**–3**′**^a^
β-actin	Forward	ACCGAGCGCGGCTACAG
	Reverse	CTTAATGTCACGCACGATTTCC
	Probe	(FAM)-TTCACCACCACGGCCGAGC-(TAMRA)
IL-8	Forward	CTGGCCGTGGCTCTCTTG
	Reverse	CCTTGGCAAAACTGCACCTT
	Probe	(FAM)-CAGCCTTCCTGATTTCTGCAGCTCTGTGT-(TAMRA)
MCP-1	Forward	CAAGCAGAAGTGGGTTCAGGAT
	Reverse	CAAGCAGAAGTGGGTTCAGGAT
TLR5	Forward	GCACTTTTATCAATTGGCTTAATCAC
	Reverse	AACGAGTCAGGGTACACACAATATATG
TNFα	Forward	GCAGGTCTACTTTGGGATCATTG
	Reverse	GCGTTTGGGAAGGTTGGA

*^a^FAM, 6-carboxy-fluorescein; TAMRA, tetramethyl-6-carboxyrhodamine*.

### Immunoblotting

Cells were stimulated for 30 min or 5 h with butyrate, propionate, or TSA, washed and lysed directly in Laemmli sample buffer (62 mM Tris/HCl pH 6.8; 2% SDS; 10% glycerol; 5% 2-mercaptoethanol; 0.01% bromophenol blue). Cell lysates were briefly sonicated, separated on 15% SDS-polyacrylamide gels and transferred to Immobilon-P polyvinylidene difluoride (PVDF) transfer membranes (0.45 μm, Millipore, Amsterdam, The Netherlands). Blots were blocked with PBS/2% milk/0.1% Tween20 and immunostained o/n at 4^°^C with a polyclonal rabbit antibody against acetylated-lysines (#9441, Cell Signaling Technology, Bioké, Leiden, The Netherlands) at a 1:1,000 dilution. Other polyclonal antibodies used were directed against acetyl-H3K9 (Merck, 07-352), acetyl-H4K5 (Merck, 07-327), acetyl-H4K16 (Merck, 07-329), HDAC1 (Abcam, ab109411), HDAC2 (Abcam, ab124974) and HDAC3 (Abcam, ab16047). As a secondary antibody, HRP-conjugated goat anti-rabbit antibody (Santa Cruz Biotechnology, Heidelberg, Germany) was used. As a loading control, GAPDH antibody (Sigma-Aldrich) was used at 1:10,000 dilution. Signal was visualized with the SuperSignal West Pico Chemiluminescent system (Thermo Scientific, Rockford, IL, USA) in a ChemiDoc MP (Biorad).

### Statistical Analysis

Statistical analysis was performed by using a paired *t*-test in Graph Pad Prism 4 software. The *t*-test can be applied to our data because each value represents a set of individual measurements. All graphs depict mean and standard error of the mean (SEM) of three independent experiments. A *p*-value of <0.05 was considered significant.

## Results

### SCFAs Modulate TLR5-Mediated NF-κB Activation

**Figure 1:**
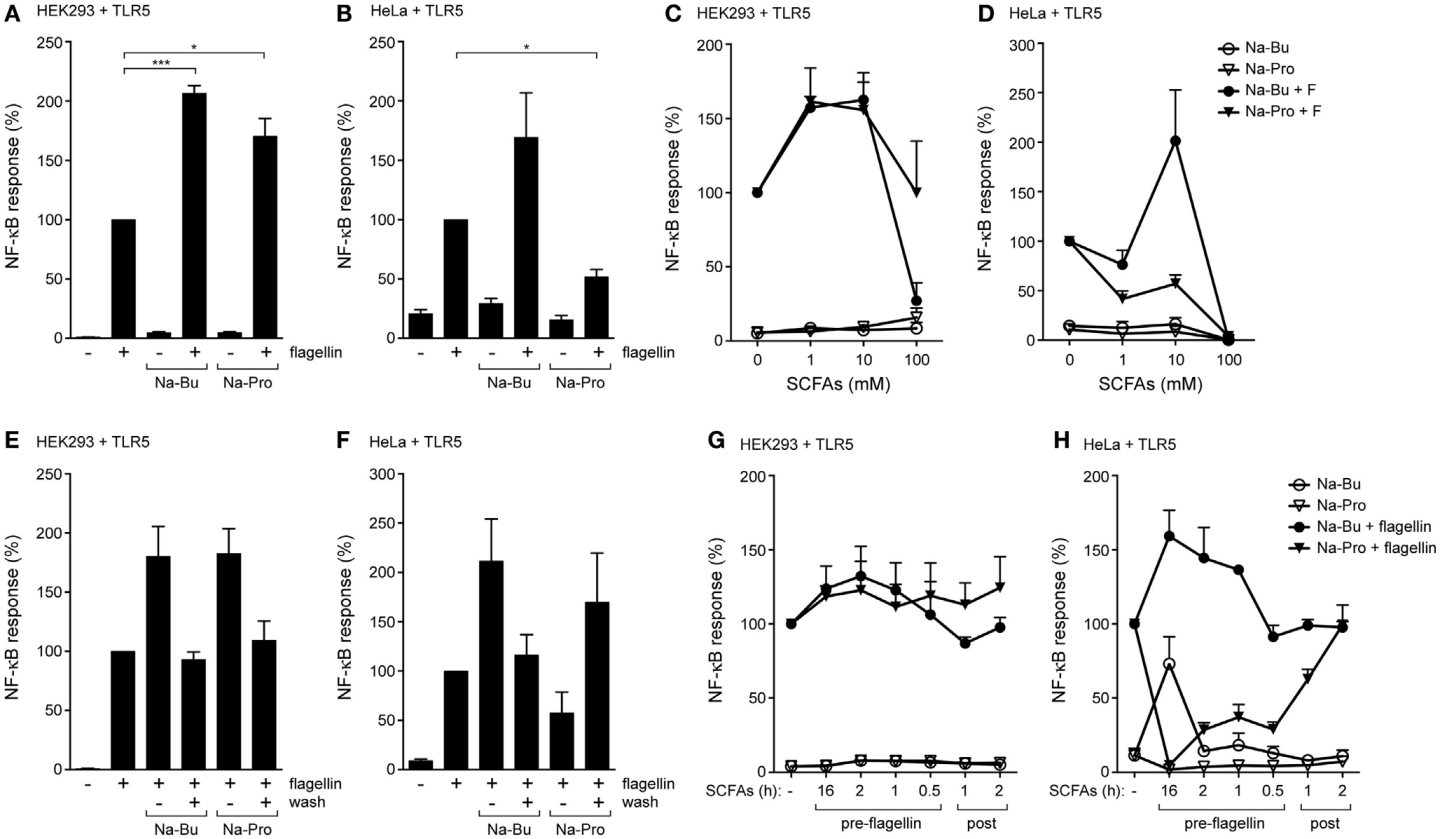
**SCFAs enhance TLR5-NF-κB responses in epithelial cells. (A,B)** TLR5-NF-κB assay in HEK293 or HeLa 57A expressing TLR5 and a NF-κB-luciferase reporter. Cells were incubated with 10 mM sodium butyrate (Na-Bu) or sodium propionate (Na-Pro) for 30 min followed by stimulation with *Salmonella enteriditis* flagellin for 5 h. **(C,D)** TLR5-NF-κB assay in HEK293 or HeLa 57A cells as described above with SCFA concentrations ranging from 0 to 100 mM. **(E,F)** Reversibility of the immunomodulatory effect of SCFAs. TLR5-NF-κB assay in HEK293 or HeLa 57A cells incubated with 10 mM Na-Bu or Na-Pro for 30 min after which SCFAs of selected groups were removed by washing. **(G,H)** TLR5-NF-κB assay to investigate pre- and post-incubation effects of SCFAs. Na-Bu or Na-Pro was added to HEK293 or HeLa 57A cells 16 h–30 min prior or 1–2 h after addition of flagellin. Bars depict mean and SEM of three independent experiments. *p* < 0.05 (*), *p* < 0.01 (**), *p* < 0.005 (***).

We set out to determine the effects of SCFAs in kidney-derived HEK293 and cervical HeLa epithelial cells because they allow efficient transfection with TLR expression and NF-κB reporter constructs. Cells were transfected with the TLR5 gene and an NF-κB dependent luciferase reporter construct and incubated with 10 mM butyrate or propionate for 30 min followed by stimulation with the TLR5 agonist flagellin for 5 h. NF-κB responses were significantly increased in HEK293-TLR5 cells pre-incubated with either butyrate or propionate (Figure 1A). In HeLa 57A cells, butyrate enhanced TLR5-induced NF-κB activation, while propionate significantly diminished the TLR5 response (Figure 1B). Administration of SCFAs alone (without TLR agonist) did not influence baseline NF-κB-luciferase values. The physiological concentrations of SCFAs in the gut range from 1 to 25 mM (5, 6). Therefore, we next tested different concentrations of butyrate or propionate to determine the dose-dependency of the observed SCFA effects. In HEK293 cells, butyrate most strongly enhanced the TLR5-mediated NF-κB activation at a concentration of 10 mM (Figures 1C,D). The enhancing effect of propionate was most pronounced between 1 and 10 mM (Figure 1C). Propionate dampened the TLR5 response in HeLa 57A cells irrespective of the tested SCFA concentration (Figure 1D); only a non-physiological SCFAs concentration of 100 mM reduced the flagellin-induced NF-κB activity, likely due to cytotoxicity. Incubation with SCFAs for 30 min followed by removal of the metabolites prior to stimulation with bacterial flagellin yielded NF-κB activity levels similar to those of flagellin alone for butyrate-treated cells (Figures 1E,F). For propionate-treated cells, a minor enhancement rather than an inhibition of the NF-κB response was observed (Figures 1E,F). These results indicate that the SCFAs need to be continuously present to elicit the enhanced TLR response.

### Kinetics of the SCFAs Modulatory Effects

We next determined the kinetics of the effects of SCFAs on TLR activation. Butyrate or propionate was added to HEK293-TLR5 or HeLa-TLR5 cells at different time points before or after the addition of flagellin. In HEK293-TLR5 cells, the strongest enhancement of TLR5 signaling was observed when butyrate or propionate was added 2 h before stimulation with flagellin (Figure 1G). In HeLa-TLR5 cells, addition of butyrate 16 h prior to flagellin stimulation led to the strongest enhancement of TLR5-mediated NF-κB activation, while addition of propionate completely abolished NF-κB activation in response to flagellin. The addition of butyrate or propionate after initiation of TLR5 signaling only moderately affected NF-κB activation in both cell types (Figure 1H).

### TLR2/1, TLR4, and TLR9 Responses are Affected by SCFAs

**Figure 2:**
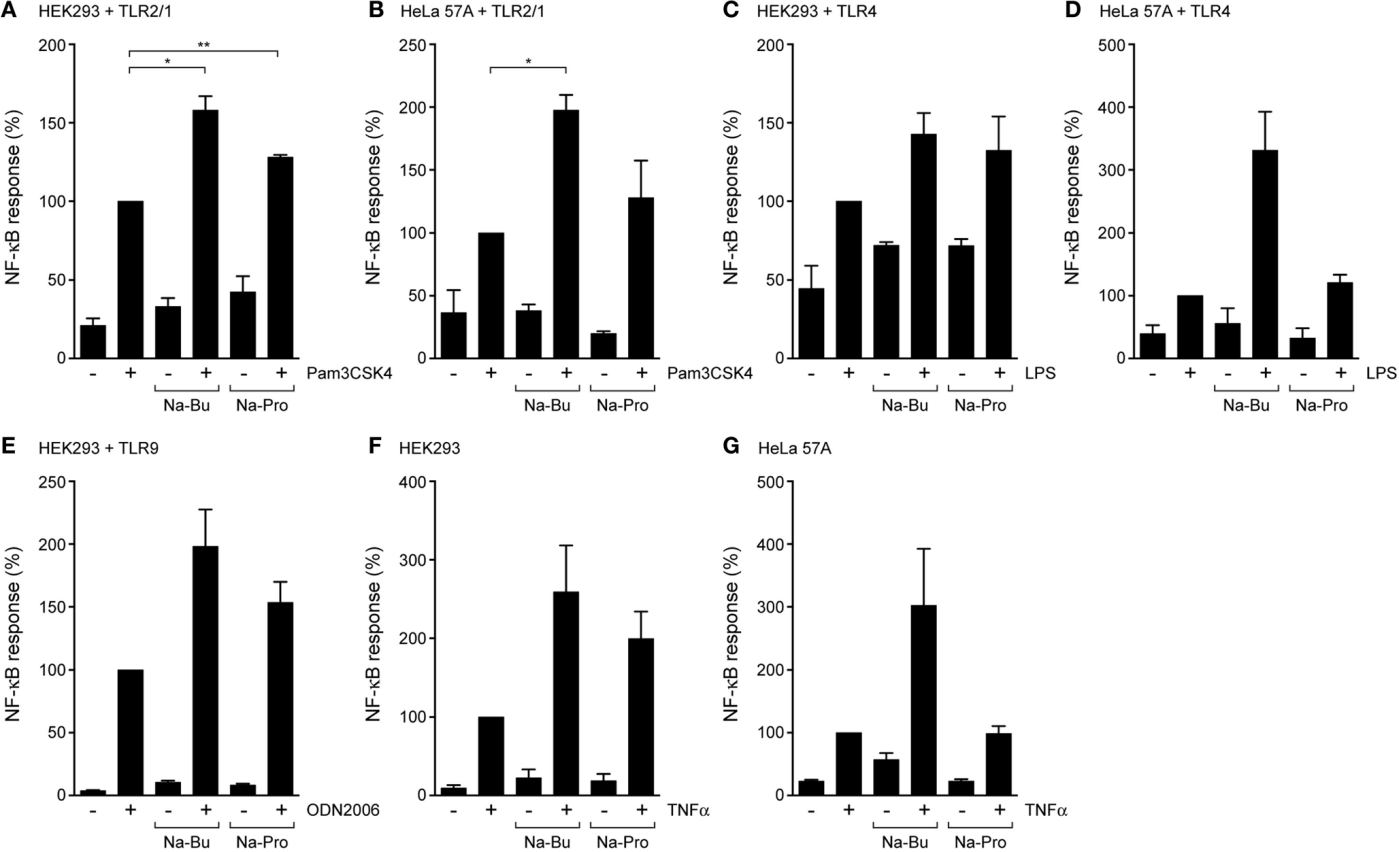
**SCFAs enhance TLR and TNFα responses in epithelial cells**. TLR–NF-κB assays in HEK293 or HeLa 57A expressing indicated TLR constructs and a NFκB-luciferase reporter. Cells were incubated with 10 mM Na-Butyrate or Na-Propionate for 30 min followed by stimulation with the respective TLR ligands for 5 h. **(A,B)** TLR2/1-mediated NF-κB activation in HEK293 or HeLa 57A cells in the presence and absence of SCFAs in response to stimulation with TLR2/1 ligand Pam3CSK4. **(C,D)** TLR4-mediated NF-κB activation in HEK293 or HeLa 57A cells in the presence and absence of SCFAs in response to stimulation with TLR4 ligand LPS. **(E)** TLR9-mediated NF-κB activation in HEK293 cells in the presence and absence of SCFAs in response to stimulation with TLR9 ligand ODN2006. **(F,G)** TNFα-mediated NF-κB activation in HEK293 or HeLa 57A cells in the presence and absence of SCFAs. Bars depict mean and SEM of three independent experiments. *p* < 0.05 (*), *p* < 0.01 (**), *p* < 0.005 (***).

To investigate whether the effects of SCFAs were specific to TLR5, we characterized TLR2/1 and TLR4 responses in the presence of butyrate and propionate using HEK293 and HeLa cells, expressing the NF-κB-luciferase reporter and different TLRs. Incubation with 10 mM butyrate for 30 min enhanced Pam_3_CSK_4_-mediated activation of the TLR2/1 complex (Figures 2A,B) and LPS-induced activation of TLR4 (Figures 2C,D) in both cell types. Propionate enhanced both responses in HEK293 cells (Figures 2A,C), but in HeLa cells these effects on the TLR2/1 and TLR4 responses were weak to absent (Figures 2B,D). This was surprising, as propionate strongly reduced NF-κB responses after stimulation of TLR5 (Figure 1). To assess whether the effect of SCFAs was limited to cell-surface exposed TLRs (TLR5, TLR2/1, and TLR4), a similar assay was performed with TLR9 that is located and functional in an intracellular compartment (22, 23). While both butyrate and propionate treatment showed a trend toward enhancing the NF-kB response in response to the TLR9 agonist ODN2006 in HEK293 cells, these differences did not reach statistical significance (Figure 2E). Overall, we conclude that incubation with SCFAs for the relatively short period of 30 min in general leads to enhancement of TLR–NF-κB activation.

### SCFAs Enhance NF-κB Activation in Response to TNFα

To investigate whether the effect of SCFAs was restricted to TLR responses, we determined their effects on the cytokine TNFα-induced NF-κB activation. In the absence of SCFAs, TNFα (5 ng/ml) activated NF-κB in both HEK293 and HeLa 57A cells. Treatment of the cells with SCFAs (10 mM) prior to TNFα incubation enhanced NF-κB responses by two- to fourfold compared to TNFα alone (Figures 2F,G). The addition of propionate to HeLa 57A cells failed to modulate TNFα-induced NF-κB levels, consistent with the results for TLR2/1 and TLR4 in these cells. Overall, these experiments show that SCFAs modulate the responses of multiple TLRs and TNFα, suggesting that the metabolites influence a common step in these inflammatory signaling cascades.

### Redirection of Cytokine Responses by SCFAs

**Figure 3:**
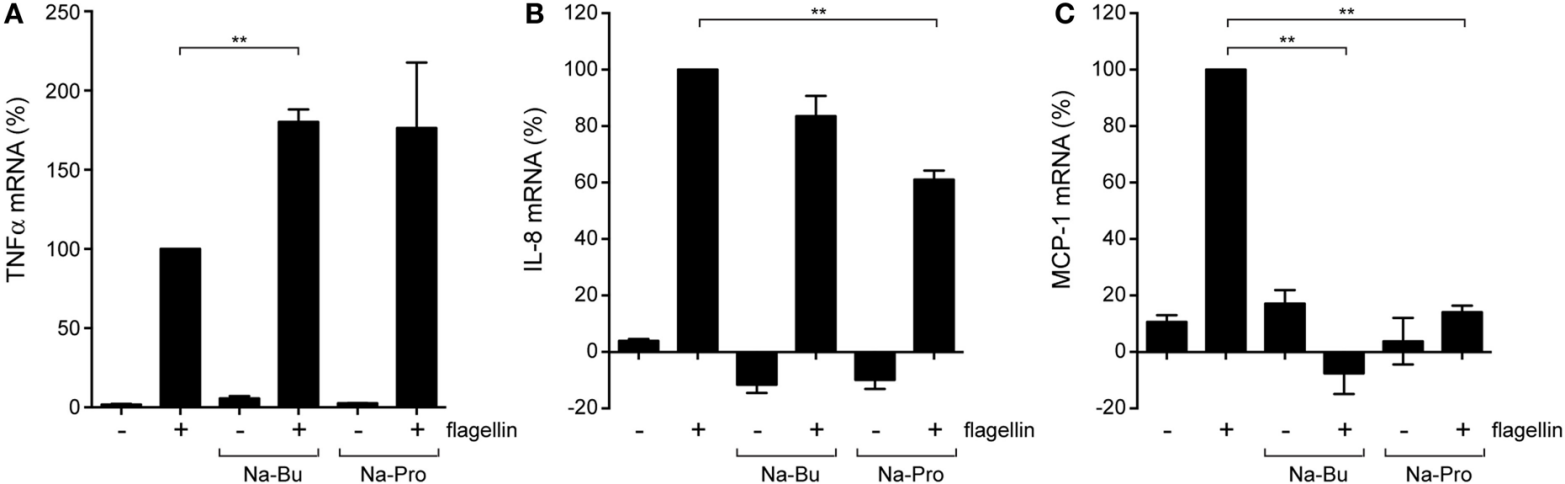
**Modulation of cytokine expression by SCFAs**. Quantitative RT-PCR of colonic epithelial HT-29 cells incubated with 10 mM Na-Butyrate or Na-Propionate for 30 min and stimulated with flagellin for 5 h. **(A)** mRNA levels of the pro-inflammatory cytokine TNFα. **(B)** mRNA levels of neutrophil chemotactic factor (IL-8). **(C)** mRNA levels of monocyte chemotactic protein 1 (MCP-1). Bars depict mean and SEM of three independent experiments. *p* < 0.05 (*), *p* < 0.01 (**), *p* < 0.005 (***).

To learn more about the downstream effects of SCFAs on intestinal TLR responses, we incubated colon epithelial HT-29 cells that endogenously express functional TLR5 with butyrate or propionate (10 mM, 30 min pre-incubation) and bacterial flagellin (200 ng/ml, 5 h incubation). qRT-PCR was used to determine changes in transcript levels for TLR5, TNFα, and the chemokines interleukin 8 (IL-8) and MCP-1 (also know as CCL2). Exposure to flagellin alone induced a strong increase in TNFα, IL-8, and MCP-1 transcript levels compared to non-stimulated cells (Figures 3A–C) and TLR5 gene expression itself was not altered by presence of the SCFAs and/or the addition of flagellin (data not shown). TNFα transcript levels were higher in the presence of butyrate and propionate, but SCFA administration dampened flagellin-induced IL-8 transcript levels and even completely abolished the upregulation of MCP-1 (Figures 3A–C). These results indicate that SCFAs can differentially alter TLR-induced inflammatory gene expression and, thus, can modulate the nature of the innate immune response.

### SCFAs Regulate the Epigenetic State of Host Cells

**Figure 4:**
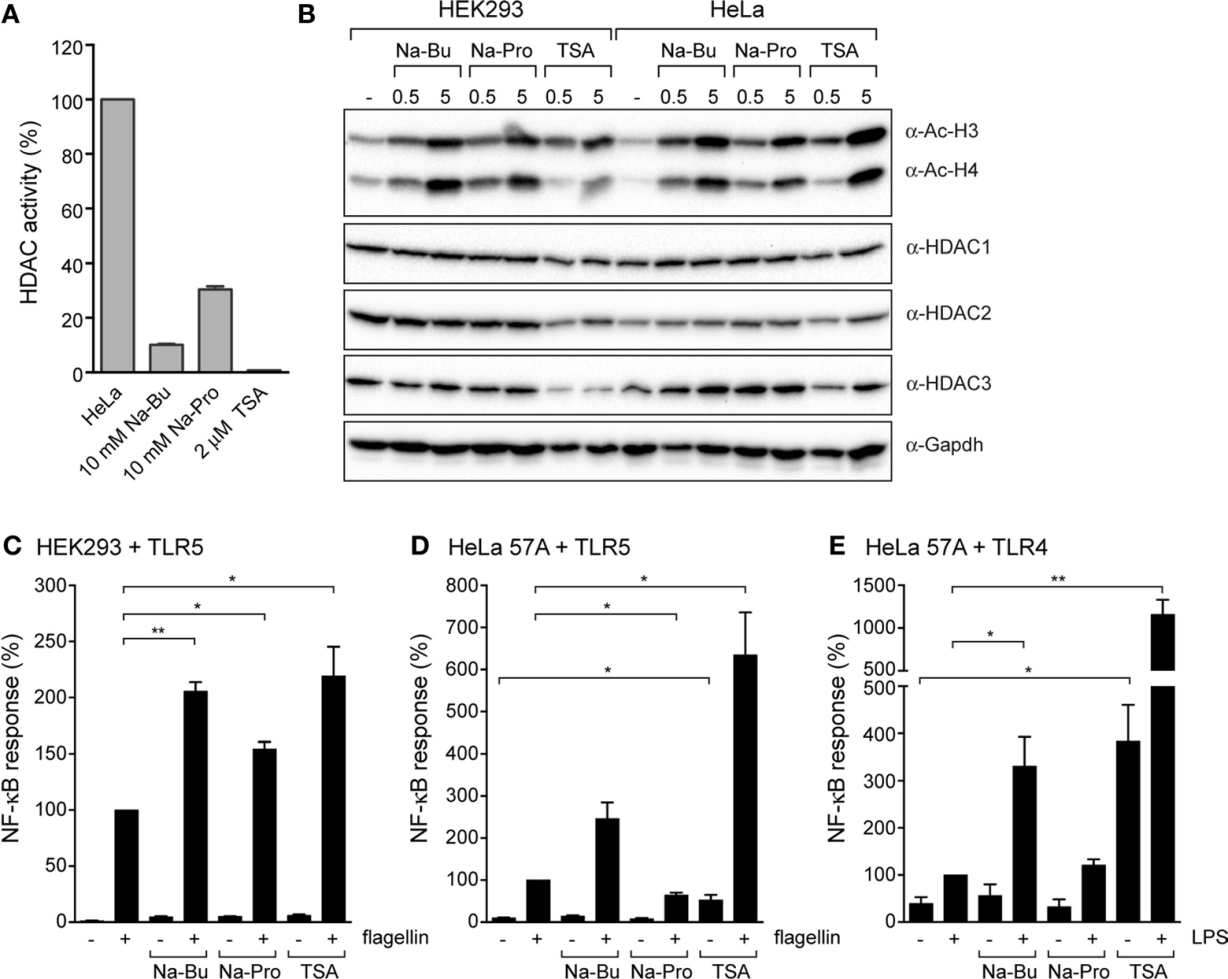
**Immunomodulatory effects of SCFAs are mimicked by the histone deacetylase inhibitor TSA. (A)** HDAC activity in HeLa lysates in the presence of 10 mM Na-butyrate, Na-propionate or 2 μM TSA. **(B)** Immunoblots of HEK293 and HeLa 57A incubated with 10 mM Na-Bu, 10 mM Na-Pro or 2 μM TSA for 30 min or 5 h analyzed with anti-acetyl-lysine antibody to visualize total histone acetylation and anti-HDAC1, HDAC2, HDAC3, and GAPDH antibodies. **(C,D)**. Immunomodulatory effects of SCFAs are mimics by histone deacetylase inhibitor TSA. TLR5-NF-κB assays of HEK293 or HeLa 57A expressing TLR5 and an NF-κB-luciferase reporter. Cells were incubated with 10 mM Na-Bu, 10 mM Na-Pro or 2 μM TSA for 30 min followed by stimulation with *S. enteriditis* flagellin for 5 h. **(E)** TLR4-NF-κB assays of HEK293 or HeLa 57A expressing TLR4 and an NF-κB-luciferase reporter incubated with SCFA and LPS. Bars depict mean and SEM of three independent experiments. *p* < 0.05 (*), *p* < 0.01 (**), *p* < 0.005 (***).

How do SCFAs exert their effects on epithelial cells? It was previously shown that SCFAs can inhibit HDACs that are involved in removal of acetyl groups from histones (24–26). Interestingly, HDAC1, HDAC2, and HDAC3 were previously shown to be negative regulators of TLR-mediated NF-κB activation (27). We performed an HDAC activity assay to directly determine the inhibitory effects of SCFAs on HDACs. HDAC activity was measured in a HeLa cell lysate without SCFAs or in the presence of butyrate (10 mM), propionate (10 mM), or the potent pan-HDAC inhibitor TSA (2 μM). All three compounds had pronounced inhibitory effects on HDAC activity; TSA led to a very strong reduction to <1% of the original activity, while incubation with butyrate resulted in 10 and 30% remaining HDAC activity, respectively (Figure 4A). Next, we determined histone acetylation levels in HEK293 and HeLa cells pre-incubated with butyrate, propionate, or TSA. Cell lysates were analyzed by immunoblot with an anti-acetyl-lysine antibody. In both cell types, incubation with butyrate, propionate, or TSA led to a marked increase of histone acetylation levels, even after a short incubation of 30 min (Figure 4B). Histone acetylation levels were further increased after a 5-h incubation with the compounds. Overall, HDAC protein levels remained unchanged (HDAC1 and HDAC2) or slightly decreased after incubation with TSA (HDAC3) (Figure 4B). Each histone has several lysines that can be modified by acetylation. We hypothesized that butyrate, propionate, and TSA may exert differential effects on individual HDACs, resulting in a difference in acetylation of specific histone residues. To investigate this, we performed immunoblot analysis using antibodies specific for acetylation of histone 3 lysine 9 (H3K9), histone 4 lysine 5 (H4K5), and histone 3 lysine 9 (H4K16). A similar increase in acetylation was observed for H3K9, H4K5, and H4K16 in response to butyrate, propionate, and TSA (data not shown). The acetylation patterns were comparable to the result observed with the general acetyl-lysine antibody (Figure 4B). Together these results convincingly show that SCFAs have rapid and profound effects on host epigenetics by inhibition of HDACs.

### Effect of HDAC Inhibition on TLR Responses

To ascertain that HDAC inhibition can contribute to the observed modulation of the TLR response by SCFAs, we compared TLR5 responses in the presence of SCFAs with the effect of TSA. In HEK293-TLR5 cells, TSA enhanced the flagellin-induced NF-κB activation to a comparable level as butyrate (Figure 4C). In HeLa–TLR5 and HeLa–TLR4 cells, TSA induced a very strong NF-κB response after stimulation with flagellin or LPS. Surprisingly, TSA by itself (in the absence of TLR ligands) was capable of inducing a significantly higher level of NF-κB activation (Figures 4D,E). Overall, these results show that SCFAs and the HDAC inhibitor TSA have similar immunomodulatory effects on TLR responses.

## Discussion

**Figure 5:**
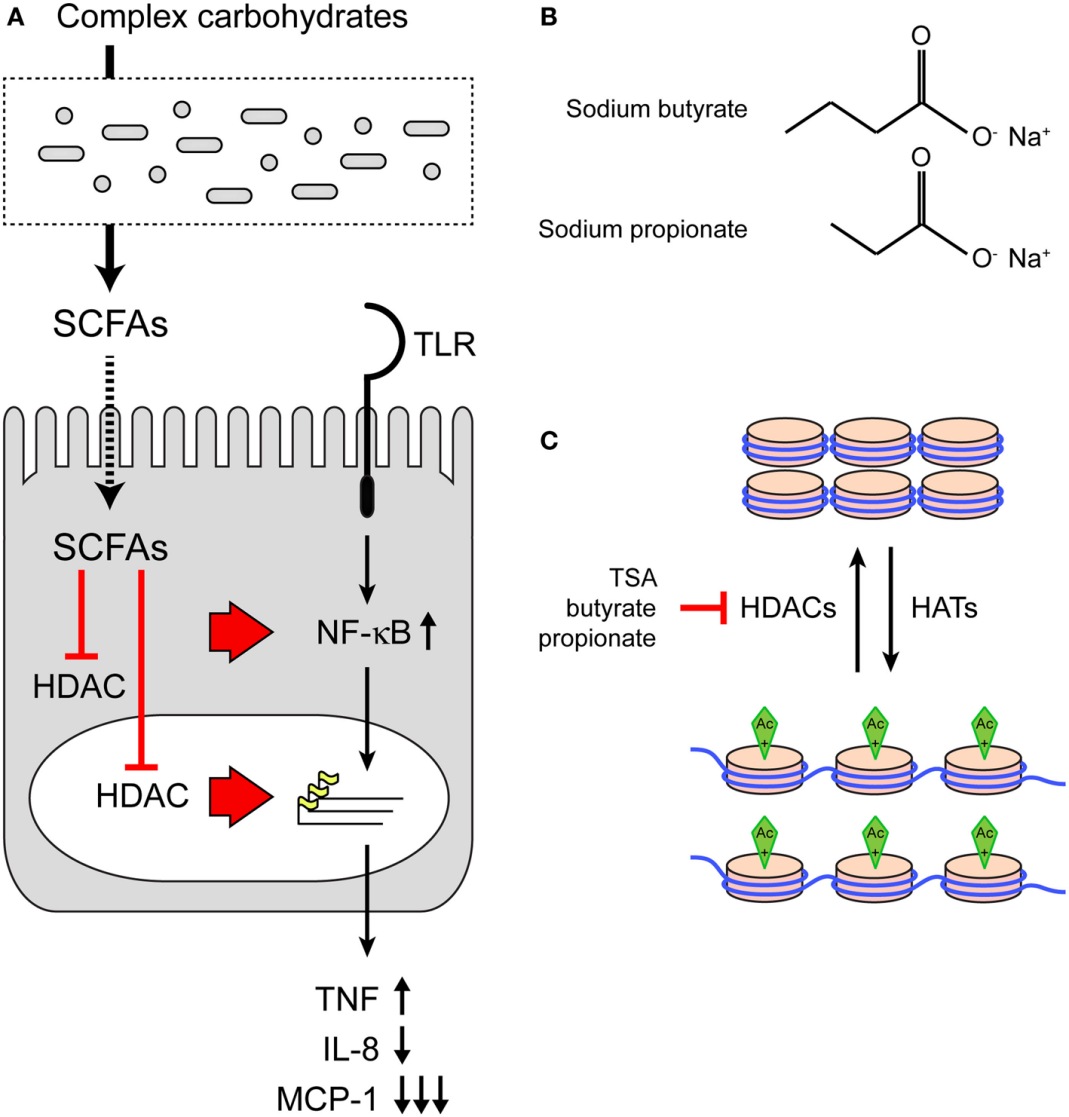
**The effect of SCFAs on HDACs and TLR responses. (A)** Model of immunomodulation of host epithelial cells by bacterial SCFAs. SCFAs are produced by microbiota from complex carbohydrates. SCFAs can inhibit HDACs in the cytosol or nucleus. Inhibition of HDACs leads to increased acetylation of histones and changes in DNA transcription. In addition, HDAC inhibition may directly impact the NF-κB pathway as the p65 subunit is also regulated by acetylation. **(B)** Structures of SCFAs sodium butyrate and sodium propionate. **(C)** The effect of SCFA on histone acetylation and chromatin structure. Top: DNA and histones in a closed chromatin structure with low levels of transcription. Acetylation by histone acetyl transferases (HATs) induces a open chromatin structure and active transcription (bottom). Histone deacetylases remove acetyl groups. Inhibition of HDACs by SCFAs leaves chromatin in the open, transcriptionally active state.

Short-chain fatty acids are produced in the intestinal lumen during the bacterial fermentation of dietary fibers and are a major cellular energy source for intestinal epithelial cells. Here, we provide evidence that SCFAs also redirect epithelial immune responses. The SCFAs butyrate and propionate enhanced NF-κB reporter activation after stimulation with various TLR ligands as well as TNFα. The modulatory effect of the SCFAs was accompanied by a change in histone acetylation and could be mimicked by the known HDAC inhibitor TSA. In colonic epithelial cells, butyrate and propionate selectively altered TNFα, IL-8, and MCP-1 gene transcription in response to stimulation with TLR ligands. In the presence of SCFAs, the pro-inflammatory cytokine TNFα is upregulated, but chemotactic chemokines IL-8 and MCP-1 are downregulated. Therefore SCFA may contribute to local containment of the intestinal inflammatory response. We conclude that bacterial SCFAs inhibit host HDAC enzymes, resulting in altered immune responses (Figure 5A).

The physiological concentrations of SCFAs in the gut lumen are 1–25 mM (5, 6). Most of our experiments were performed with a 10 mM SCFA concentration, well within the physiological concentration. By addition of SCFAs 30 min before TLR stimulation, we mimicked the temporal spike of SCFA levels after a meal. The direct, rapid effects on TLR signaling and cytokine production that we observe, therefore, differ from the well-studied long-term effects of Na-Bu on cell proliferation and differentiation. These long-term effects are generally observed after more than 24 h exposure to SCFAs. Our results suggest that transient changes in SCFA concentrations in the gut can also redirect the nature of the innate immune response.

The pleiotropic effects of SCFAs have been under intense investigation since the 1970s when several groups reported effects of Na-Bu on cell proliferation, differentiation, and gene expression. Following these observations, it was established that butyrate inhibits HDACs, resulting in increased histone acetylation (24–26) (Figure 5C). SCFAs such as butyrate and propionate are non-competitive pan-HDAC inhibitors (28). The crystal structure of an HDAC-like protein with the HDAC inhibitor TSA demonstrates that TSA occupies a hydrophobic cleft on the surface of the enzyme away from the substrate-binding site (29). The binding site of butyrate on an HDAC enzyme is still not identified, but it is possible that two molecules of butyrate may bind the same hydrophobic cleft (30). Considering the potent effects of butyrate on HDACs and histone acetylation, it is surprising that the expression of as little as 2% of mammalian genes is affected in the presence of butyrate (31, 32).

We show that a short incubation with SCFAs is sufficient to sensitize NF-κB activation by TLR5, TLR2/1, TLR4, and TLR9. In addition, SCFAs also increased NF-κB activation in response to incubation with TNFα. Surprisingly, SCFAs even induce low-level NF-κB activation in absence of TLR ligands (Figures 1H and 2G). Together these data indicate that SCFAs most likely modify a common target in the NF-κB pathway rather than modulate a particular signaling component. We hypothesized that SCFAs exert their effects on NF-kB signaling by inhibition of HDACs and altering chromatin structure. We tested this hypothesis by comparing the effects of SCFAs and the strong HDAC inhibitor TSA on histone acetylation and NF-κB activation in response to TLR ligands (Figure 4). Incubation with butyrate, propionate, and TSA lead to rapid changes in histone acetylation (Figure 4B). These results are in line with the reported effects of these compounds in other systems (33, 34). In addition, TSA was very potent at sensitizing TLR responses and NF-κB was even activated in the absence of TLR ligand (Figures 4C–E). These data convincingly show that HDAC inhibition leads to sensitization of NF-κB responses. The rapid increase in SCFA-induced histone acetylation (30 min) corroborates the rapid alterations in TLR responses (Figures 1–3). These results together strongly support the notion that the observed effects of SCFAs on epithelial immune responses are caused by inhibition of HDAC enzymes, resulting in changes in histone acetylation.

The structures of Na-Bu (C4) and Na-Pro (C3) are very similar, but it is well established that HDAC inhibition by butyrate is stronger than inhibition by propionate (Figures 5B,C). The HDAC assay that we performed also shows that TSA is the strongest HDAC inhibitor, followed by butyrate and propionate (Figure 4A). These differences in HDAC inhibition are nicely recapitulated in a TLR–NF-κB activation assay, where TSA has the strongest sensitizing effect followed by butyrate and propionate (Figures 4C–E). The effect of butyrate is consistently stronger than the effect of propionate (for example, Figures 1A,D and 2B). Interestingly, we observed that butyrate and propionate have completely opposite effects in a time course of SCFA–TLR ligand addition in HeLa but not in HEK293 cells (Figures 1G,H). Incubation with butyrate enhanced NF-κB responses, while propionate completely dampened the response. SCFA specificity or different levels of HDAC expression or accessibility may cause these opposite effects. The human HDAC family consists of 11 proteins with distinct expression patterns, subcellular localization, target proteins, and protein sites. Protein expression of HDAC1, HDAC2, and HDAC3 was comparable after incubation with butyrate and propionate, while expression of HDAC3 was reduced after incubation with TSA (Figure 4B). It is conceivable that butyrate, propionate, and TSA inhibit the individual HDACs to a different extent, resulting in tailored epigenetic regulation.

Our results fit well with a body of work that establishes class I histone deacetylase enzymes HDAC1, HDAC2, and HDAC3 as negative regulators of TLR pathways [reviewed by Shakespear et al. and Falkenberg and Johnstone (27, 35). For example, HDAC1 inhibits pro-inflammatory gene promoters of IL-12p40, Cox-2, and Ifn-β (36–38). Activating transcription factor 3 (ATF3) and metastatic tumor antigen 1 (MTA1) are downstream targets of TLR signaling, which are regulated by HDAC1 and HDAC2, respectively (39, 40). Regulation of MTA1 by HDAC2 prevents activation of inflammatory cytokine genes TNFα and IL-1β (40). HDAC1, HDAC2, and HDAC3 are well expressed in our epithelial cell lines (Figure 3B). It is, therefore, conceivable that the immunomodulatory effects of the SCFAs in our study are caused by inhibition of these three enzymes. Besides histone targets, HDACs also deacetylate other proteins. The p65 subunit of NF-κB itself is acetylated and p65 deacetylation by HDAC3 leads to association with Iκβa, nuclear export, and dampening of the NF-κB response (41). It is, therefore, possible that in addition to histone modification, SCFAs alter acetylation of NF-κB subunits themselves. It remains to be established whether the effects of SCFAs on epithelial immune responses are caused by changes in histone acetylation, direct modification of NF-κB subunits, or both.

Little is known about the role of individual HDACs in deacetylation of specific lysines in histones. It was shown that HDAC inhibition by Na-Bu leads to an increase in acetylation of lysine 9 of histone 3 (H3K9) in mouse subventricular cells (42). Mouse embryo HDAC1 knockout cells show an increase in acetylation of lysine 5 of histone 4 (H4K5) (43). Acetylation of lysine 9 of histone 3 (H4K16) is a very specific acetylation event that is regulated by class III HDACs, also known as Sirtuins (44). We investigated the impact of SCFAs on H3K9, H4K5, and H4K16 histone acetylation with acetylation-specific antibodies. To our surprise, H3K9, H4K5, and H4K16 acetylation patterns were comparable to the pattern observed with the general acetyl-lysine antibody (Figure 4B, data not shown). These results indicate that SCFAs and TSA have a general HDAC inhibitory effect on all HDACs involved in deacetylation of H3K9, H4K5, and H4K16 and differences in acetylation might be too subtle to detect with this method.

One of our most striking observations is that a 30-min incubation with butyrate or propionate enhanced the TLR-induced TNFα expression but dampened or even abolished IL-8 and MCP-1 transcription in colonic HT-29 cells (Figure 3). The pro-inflammatory cytokine TNFα is an essential mediator of inflammation in the intestine. IL-8, also known as neutrophil chemotactic factor, and MCP-1 are important in the recruitment of immune cells to the site of activation. Our results, therefore, indicate that SCFAs enhance local pro-inflammatory responses, but dampen the recruitment of inflammatory cells to the site of infection. Modulation of the epithelial cytokine response by SCFAs seems to be cell type and condition dependent, as it was previously shown that neutrophil migration toward Caco-2 is decreased after overnight incubation with SCFAs and increased toward HT-29 (11). In addition, butyrate enhances IL-8 secretion by Caco-2 cells in response to IL-1β and LPS (45). SCFAs also have anti-inflammatory effects on cytokine secretion by endothelial cells, macrophage and neutrophils (46). Because of the complex time and cell-type-dependent effects of SCFAs, it is not possible to draw a general conclusion about the impact of SCFAs on cytokine responses. However, the tuning of the local TLR response by SCFAs, resulting in containment of inflammatory response, seems in line with the supposed generally anti-inflammatory role of SCFAs. For example, butyrate has been demonstrated to dampen the NF-κB pathway in intestinal biopsy samples and to decrease the production of pro-inflammatory cytokines by peripheral blood mononuclear cells (PBMCs) (47). Based on our results, we pose that bacterial SCFAs sensitize local epithelial TLR responses and prevent large-scale inflammation by selective epigenetic modification of innate immune genes.

## Conflict of Interest Statement

The authors declare that the research was conducted in the absence of any commercial or financial relationships that could be construed as a potential conflict of interest.

## Abbreviations

HDAC, histone deacetylase; IBD, inflammatory bowel disease; SCFA, short-chain fatty acids; TLR, toll-like receptors; TSA, trichostatin A.
